# Anatomical structures of fine roots of 91 vascular plant species from four groups in a temperate forest in Northeast China

**DOI:** 10.1371/journal.pone.0215126

**Published:** 2019-05-01

**Authors:** Hongfeng Wang, Zhengquan Wang, Xueyun Dong

**Affiliations:** 1 School of Forestry, Northeast Forestry University, Harbin, China; 2 School of Science, Harbin University, Harbin, China; University of Massachusetts Amherst, UNITED STATES

## Abstract

Fine roots of plants play an important role in terrestrial ecosystems. There is a close association between the anatomical characteristics and physiological and ecological functions of plants, but we still have a very limited knowledge of anatomical traits. For example, (1) we do not know if herbs and grasses have anatomical patterns similar to those of woody plants, and (2) the variation among different woody plants in the same ecosystem is unclear. In the present study, we analysed the anatomical structures of the fine root systems of various groups of vascular plants (ferns, eudicot herbs, monocots and woody plants) from the same ecosystem (a natural secondary forest on Mao'er Mountain, Heilongjiang, China) to answer the following questions: (1) How does the anatomy of the fine roots change with root order in various plant groups in the same ecosystem? (2) What is the pattern of variation within group? The results show that anatomical traits can be divided into 3 categories: traits that indicate the root capacity to transport resource along the root (stele diameter, xylem cell diameter and xylem cell area); traits that indicate absorptive capacity cortical thickness, (the number of cortical cell layers and the diameter of cortical cells); and traits that are integrated indicators (diameter and the stele to root diameter ratio). The traits indicate the root capacity to transport resource along the root order is generally similar among groups, but absorptive capacity is very different. The shift in function is the main factor influencing the fine root anatomy. Some traits show large variation within groups, but the variations in other traits are small. The traits indicate that the lower-order roots (absorbing roots) in distinct groups are of the first one or two root order in ferns, the first two or three orders in eudicot herbs, the first (only two root orders) or first two orders (more than three root orders) in monocots and the first four or five root orders in woody plants and the other roots are higher-order roots (transport roots). The result will helpful to understand the similarities and differences among groups and the physiological and ecological functions of plant roots.

## 1. Introduction

Owing to unique trait patterns [1, 2], more than 22% of the net primary productivity of global terrestrial ecosystems is transferred below ground annually via fine root turnover [3], even though the fraction of live fine root biomass to total tree biomass only ranges from less than 1% in mature forests to over 15% in young forests [4].Recognizing that there is a close association between anatomical characteristics and physiological and ecological functions, researchers have for many years classified fine roots on the basis of anatomical traits [5]. Since then, additional details have been revealed that have allowed us to understand many physiological functions and ecological processes through anatomical traits.

The anatomical structure of fine roots is thought to be closely associated with their function [6–10]. For example, a high correlation was observed between hydraulic properties and the structural properties of the transmitting tissue [10]. The characteristics of the cortex and stele may reflect the two functions of absorption and transport [7, 11].

Most plants have hierarchical root systems, with roots at distinct positions being designated as distinct root orders [2]. An anatomical study suggested that the 1^st^-order roots of woody plants mainly perform absorption, while the roots higher than the 4^th^ order greatly reduced this function [7]. In fact, the transition of the physiological functions always associated with the secondary development such as the loss of cortex, increased suberization, development of cork periderm, endodermis and thicker stele [7, 12, 13]. These reduce the ability of the root to absorb water and nutrients [14] and imply that the higher-order roots play more important roles in transport. And the fine root can be divided into absorptive and transport fine roots [3, 15].

Anatomical traits also affect long term function. Diameter has a strong relationship with lifespan [16, 17]. Therefore, anatomical traits dramatically influence the turnover of materials. The fine roots adapt to environmental changes by altering their anatomical characteristics. For example, When the environment changes, the cortex, stele and other anatomical traits also change to adapt to the new conditions [18].

However, we still have a limited knowledge of anatomical traits. For example, (1) the biomass of herbs and grasses can account for nearly 50% of the total biomass of forests [19], but we do not know if the anatomical pattern of these plants is similar to that of woody plants. (2) The variations among different woody plants in the same ecosystem are also unclear because most studies are conducted in forestry stands, which are typically monocultures [20, 21].

If these questions remain unsolved, attempts to thoroughly understand the physiological functions and ecological consequences of plant roots will be hampered. In the present study, we analyse the anatomical structure of the fine root systems of various groups of vascular plants from the same ecosystem (a natural secondary forest on Mao'er Mountain) to answer the following questions: 1. How does the anatomy of the fine roots change in various plant groups in the same ecosystem? 2. What is the pattern of variation within group?

In previous studies of plant anatomy and function, a large amount of evidence has revealed substantial differences between groups of herbaceous monocots, herbaceous eudicot, woody plants and ferns, but there is considerable consistency within each of these four groups [22]. Therefore, we hypothesized that the patterns of anatomical traits in fine roots are highly consistent within the groups of herbaceous monocots, herbaceous eudicot, woody plants and herbaceous ferns but significantly different among them.

## 2. Materials and methods

### 2.1 Site description

The study was conducted at the Mao'er Mountain Experimental Station (45°21′–45°25′N, 127°30′–127°34′E) of Northeast Forestry University in north-eastern China. The site has a continental temperate monsoon climate with mean January, July and annual air temperatures of −19.6°C, 20.9°C and 2.8°C, respectively, and the mean annual precipitation was 723 mm with 477 mm falling from June to August [7]. The soils were Hap-Boric Luvisols that were well drained with high organic matter and frozen to a depth of 1 m during the winter (from December to April). The stand was a secondary forest regenerated from a mixed temperate old-growth forest that was harvested over 70 years ago [3].

### 2.2 Experimental materials

All plant materials were collected from the understory and edge of a natural secondary forest in Mao'er Mountain National Forest Park (Shangzhi, Heilongjiang, China). We selected 8 species of herbaceous monocots (2 species were hydrophytes), 51 species of herbaceous eudicot (3 species were hydrophytes), 9 species of ferns and 23 species of woody plants. Thus, 91 species belonging to 84 genera and 38 families were included in the analysis (S1 Table) [23]. In natural secondary forests, plant roots are mixed together. When sampling, we dig down along the trunk and thicker roots. Dig and remove the soil around the root system, and look for suitable lateral roots at a depth of about 20 cm. The Suitable lateral roots are roots thicker than the fine roots, but the next lower order are fine roots (<5 mm). Carefully digging around the lateral root, we ensured that the entire lateral root was dug. We use hands, screwdriver, or small brush to carefully remove large clods from the lateral roots and then we assessed whether the lateral roots are intact. Three to 20 intact lateral roots were fully excavated for each woody species. Herbaceous plants have three types of root system: tap root system, fibrous roots system and roots formed mainly by rhizomes. For herbaceous plants with tap root system and fibrous roots system, we dig out the entire root system and carefully remove the larger clods and assess whether the root system is intact. For herbaceous plants that are vegetatively propagated through rhizomes, the rhizomes are cut off, the plant is divided into a single plant or a cluster, and then the entire root system formed by the rhizomes is excavated.

In august of 2012 5 to 10 individuals of each species were randomly sampled. Then the root was dipped in water. After removal of most soil by running water, lateral roots were dissected. The partial root system was put in a culture dish and carefully cleaned using a little brush while observing through a magnifying glass. Individual roots were dissected by root order and then cleaned with deionized water and immediately fixed in a formalin-acetic acid-alcohol (FAA) (90 ml 70% ethanol, 5 ml 100% glacial acetic acid, 5 ml 37% methanol) for anatomical analysis.

### 2.3 Anatomical analysis

We define a “fine root” as roots less than 5 mm in diameter. The roots were then divided into different branch orders following the procedure described by Pregitzer et al. (2002). For each species, 30 root segments were randomly chosen from different plants for the 1^st^ order, and 20 segments were chosen for all the other orders. All the root segments were stained with safranine-fast green (2%), dehydrated in 70, 85, 95 and 100% alcohol and then embedded in paraffin; then, 8-μm sections were mounted on glass slides [7]. For root segments less than 1-cm long, three sections near the root base (the branching point) were chosen. For root segments greater than 1 cm long, three sections were chosen that were evenly distributed between 1 cm from the root tip (for the 1^st^ order roots) or the branching point (for higher-order roots) to the root base [7, 24]. The slides were photographed (Leica, DFC540c, Germany) under a compound microscope (BH1, Olympus Corporation, Tokyo, Japan).

### 2.4 Data analysis

For each transverse slice of the roots, a range of anatomical features including root diameter, cortical thickness, stele diameter, xylem cell diameter (this is tracheids for gymnosperms and pteridophytes and vessels for the other groups), xylem cell area, diameter of the cortical cells, and number of cortical cell layers were measured to the nearest 1 μm [24] using Motic Images Advanced 3.2 software (Motic Corporation, Zhejiang, China) [23]. The “tracheids” was often present in gymnosperms and pteridophyte.

The mean, standard deviation and coefficients of variation of root morphological, histological and chemical characteristics were calculated for each root order by species. Fisher’s LSD test (P = 0.05) was used to test the differences in the root characteristics among the orders for each species when the data pass the checking for normal distribution and rank sum test (P = 0.05) was used when the data did not pass the checking. Simple regression analysis was used to determine the relationships among traits. All statistical analyses were performed using SPSS software (V. 19.0, IBM Corp., USA, 2010).

### 2.5 Ethics statement

The “Mao'er Mountain Experimental Station” is a subordinate organization to “Maoer Mao'er Mountain teaching area” of Northeast Forestry University. The study was approved by Chang song Li, the director of Maoer Mao'er Mountain teaching area.

## 3. Results

### 3.1 Differences among and within groups

**Fig 1:**
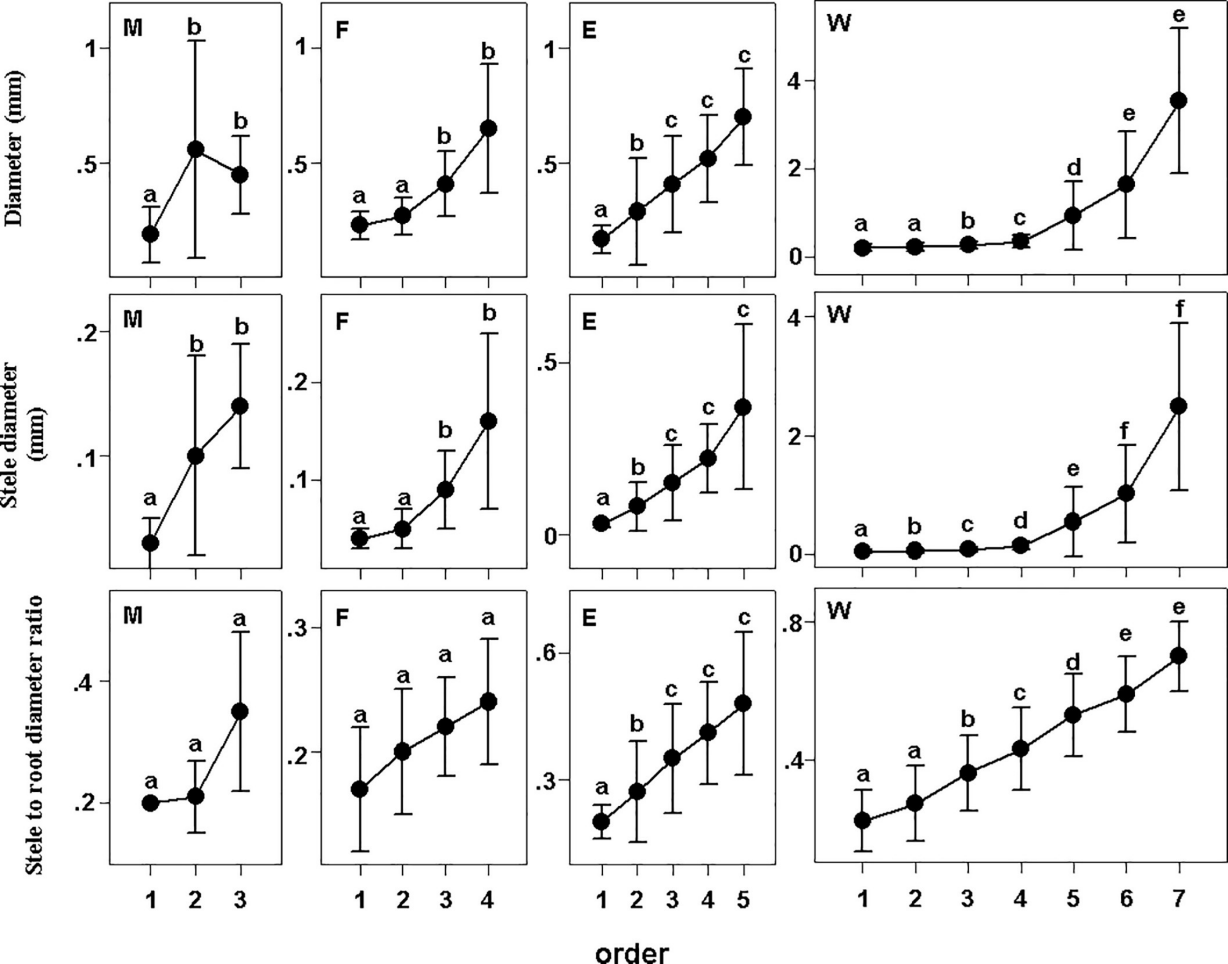
Diameter, stele diameter, xylem cell diameter, and the stele to root diameter ratio in different groups. Different lower-case letters indicate significant differences (P<0.05) among orders. The error bars represent 1 SD of the mean. M: Monocots; F: Fern; E: Eudicot herbs; W: Woody plants.

**Fig 2:**
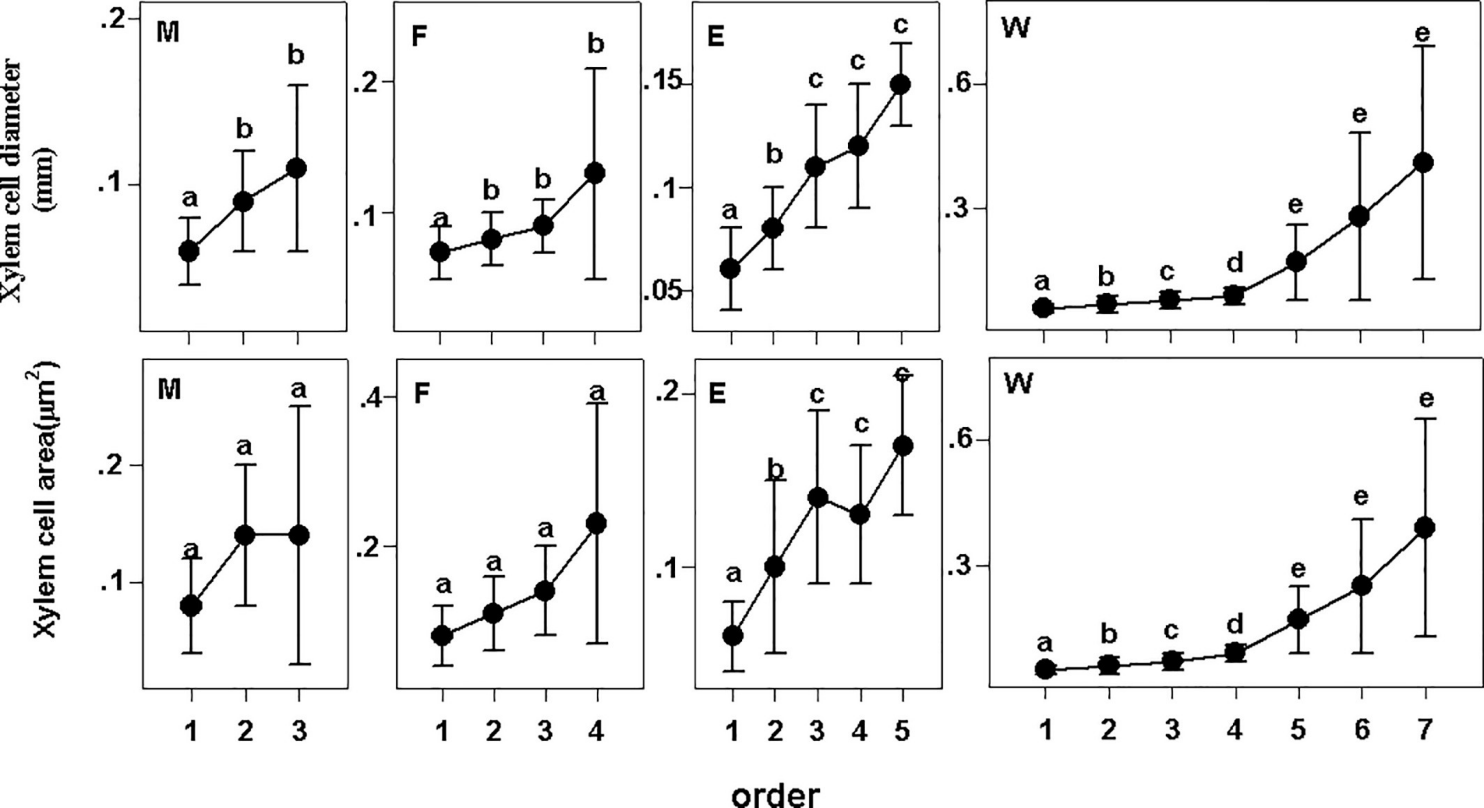
Xylem cell diameter and xylem cell area of fine roots in plant groups. Different lower-case letters indicate significant differences (P<0.05) among orders. The error bars represent 1 SD of the mean. M: Monocots; F: Fern; E: Eudicot herbs; W: Woody plants.

There were some similarities among groups as diameter, stele diameter, xylem cell diameter and xylem cell area increased with increasing root order in all groups (Figs 1 and 2). The diameter of fine roots was found to increase with increasing root order in all the examined plant groups. Stele diameter gradually increased with increasing root order in the examined groups also.

There were also some differences among and within groups. There was little difference in the diameter, stele diameter and stele to diameter ratio among orders in monocots (Fig 1). The diameter and stele diameter of the 1^st^ order roots were slightly lower than those of the 2^nd^ to 3^rd^ orders, and the difference in stele to diameter ratio was not significant in monocots (Fig 1, [23]). In eudicot herbs, the diameter of the stele was smallest (34.9 μm) in 1^st^-order roots, and the stele to diameter ratio of the 4^th^ to 5^th^ orders (0.41, 0.47) was higher than that of the 1^st^ to 3^rd^ orders (Fig 1). The 3^rd^ and 4^th^ order had the highest cortical thickness in eudicot herbs, but this does not indicate that the diameter will "shrink" in the 5^th^ orders. Instead, some plants had only 3 or 4 orders of fine roots with a large diameter. The 2^nd^ order had the highest diameter in ferns for the same reason. Diameter, stele diameter and stele to diameter ratio followed similar patterns in woody plants, but there were no significant differences in stele to diameter ratio among the 5^th^ to 7^th^ order roots. The 1^st^ orders had the smallest stele to diameter ratio (0.22), and the stele to diameter ratio of the 5^th^ to 7^th^ orders was largest in woody plants (0.52–0.70). The diameter of the 2^nd^ to 7^th^ orders differed significantly between adjacent orders (224 μm to 3516 μm), but the diameter of 1^st^ to 4^th^ was similar. Some roots, such as those of the 5^th^ to 7^th^ orders of woody plants, showed strong within-group variation. The main reason for this phenomenon was that most plants had only or less than 5 root orders; only 12 species had 6^th^ order roots, and 3 species had 7^th^ order roots.

The xylem cell diameter and xylem cell area exhibited a pattern like stele diameter, a larger stele diameter always means a larger xylem cell diameter and xylem cell area (Fig 2). However, there were some remarkable differences between xylem cell diameter and xylem cell area among them. Despite the larger stele diameter than 1^st^ and 2^nd^ order, the 3^rd^ and 4^th^ order tracheids area of ferns was not significantly different from those of the 1^st^ and 2^nd^ order. Second, the variation in xylem cell area in eudicot herbs was higher than that in xylem cell diameter and stele diameter. (Fig 2). In cross section, xylem cells were not round but greatly varied after forming the secondary wall. Thus, a similar diameter does not always mean a similar area. There were so many life forms among eudicot herbs, so the shape of the xylem cell varies, which makes the coefficient of variation large.

**Fig 3:**
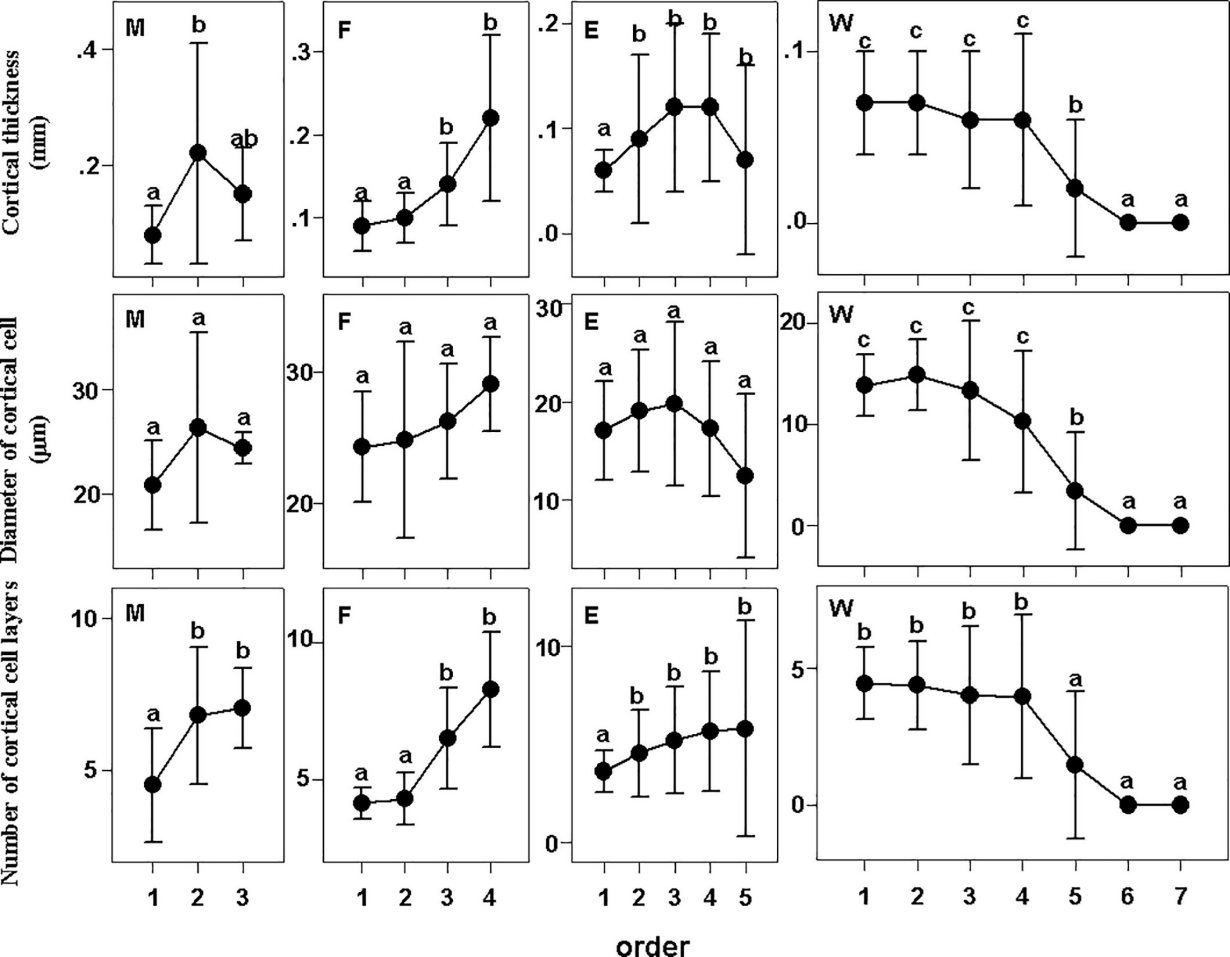
Cortical thickness, diameter of cortical cells and number of cortical cell layers of fine roots in different groups. Different lower-case letters indicate significant differences (P<0.05) among orders. The error bars represent 1 SD of the mean. M: Monocots; F: Fern; E: Eudicot herbs; W: Woody plants.

The cortex patterns were very different among groups (Fig 3). With increasing root order, the cortex becomes thicker in ferns but thinner in woody plants, while it first becomes thicker and then thinner in monocots and eudicot herbs (Fig 3, [23]). There was no significant difference in the patterns of diameter of the cortical cells, number of cortical cell layers or cortical thickness in woody plants. The characteristics of number of cortical cell layers and cortical thickness in monocots were similar, but there was no significant difference in diameter of the cortical cells between orders. With increasing root order, the cortical thickness of eudicot herbs first increased and then decreased, but the number of cortical cell layers steadily increased. There was no significant difference among orders in diameter of the cortical cells or among the 3^rd^ to 5^th^ orders in number of cortical cell layers. The variation in diameter of the cortical cells was large in monocots with no significant difference among orders, but the number of cortical cell layers increased with increasing root order (Fig 3, [23]).

### 3.2 Correlations between anatomical parameters

**Fig 4:**
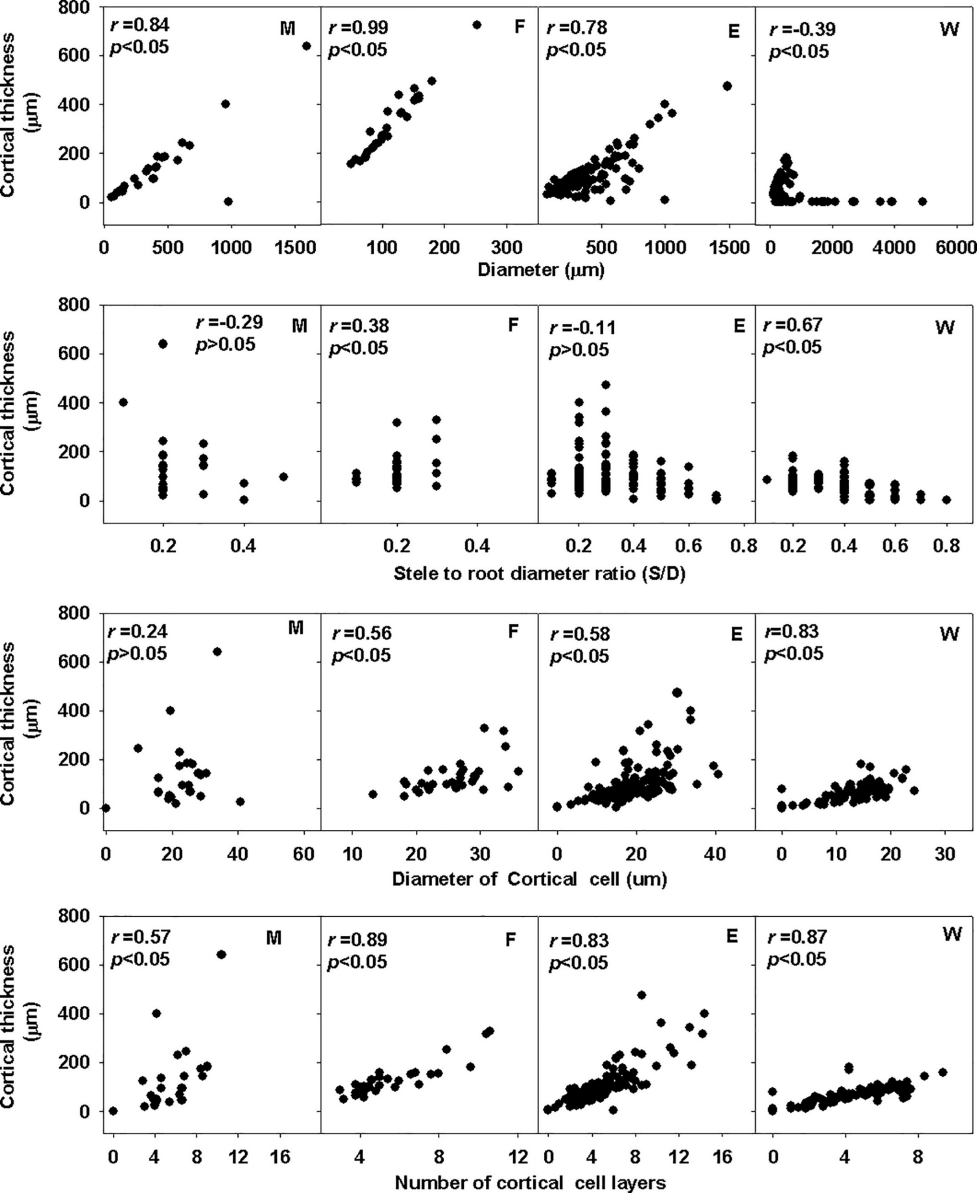
Correlation among anatomical traits in different groups. M: Monocots; F: Fern; E: Eudicot herbs; W: Woody plants. r: Pearson Correlation Coefficient p: p-value; Each point is root order in distinct groups.

Except for woody plants, the other groups showed a high correlation between cortical thickness and diameter (Fig 4). Woody plants exhibited a high correlation between cortical thickness and stele to diameter ratio, but the correlation was substantially lower in the other three groups. Cortical thickness presented a weak correlation with diameter of the cortical cells in monocots, but these two parameters were highly correlated in the other three groups. Cortical thickness was highly correlated with the number of cortical cell layers in all groups. The correlations obtained with linear regression analysis between cortical thickness and number of cortical cell layers were reasonably similar among the groups (Fig 4 bottom row). However, the correlation between cortical thickness and diameter of the cortical cells showed large differences between the various groups, being high for woody plants, low for monocots eudicot herbs and ferns (Fig 4, third row).

Monocots, eudicot herbs and ferns [23] showed a high correlation between cortical thickness and diameter, but the correlation was significantly lower in woody plants. However, woody plants exhibited a high correlation between cortical thickness and stele to diameter ratio, but no correlation was found between these parameters in the other groups (Fig 4).

## 4. Discussion

### 4.1 Similarities and differences in the anatomical structure of fine roots among various plant groups

The fine roots of different groups presumably perform similar functions, and their anatomical structures show a similar pattern. However, due to differences in the cells, life forms and other characteristics, there were also remarkable variations among and within groups.

Anatomical traits can be divided into three categories: traits that indicate the root capacity to transport resource along the root (stele diameter, xylem cell diameter and xylem cell area), traits that indicate absorptive capacity (cortical thickness, number of cortical cell layers and diameter of the cortical cells) and traits that are integrated indicators (diameter and stele to diameter ratio).

With increasing root order, the fine roots require an increased capacity for axial transport [25], so the transport function of fine roots was presumably enhanced in all plant groups [2, 7]. Therefore, traits related to the transport function (stele diameter, xylem cell diameter and xylem cell area) increase with increasing root order.

Traits related to the absorption function (cortical thickness, number of cortical cell layers and diameter of the cortical cells) were very different among groups. The cortical thickness, number of cortical cell layers and diameter of the cortical cells of higher-order roots decrease markedly in woody plants [7, 26, 27], which was significantly different from what occurs in ferns [23], monocots and herbaceous eudicots [28].

Given the negative correlation between the radial and axial transport capacity of roots, there is apparently an increasing need to reduce radial transport and thereby ensure axial transport with increasing root order [29, 30]. But plants can acquire this capacity via different pathways. In the higher-order roots of woody plants, a secondary structure (eg. periderm) generally forms [22, 25], making that formation of a thick cortex unnecessary [31].

**Fig 5:**
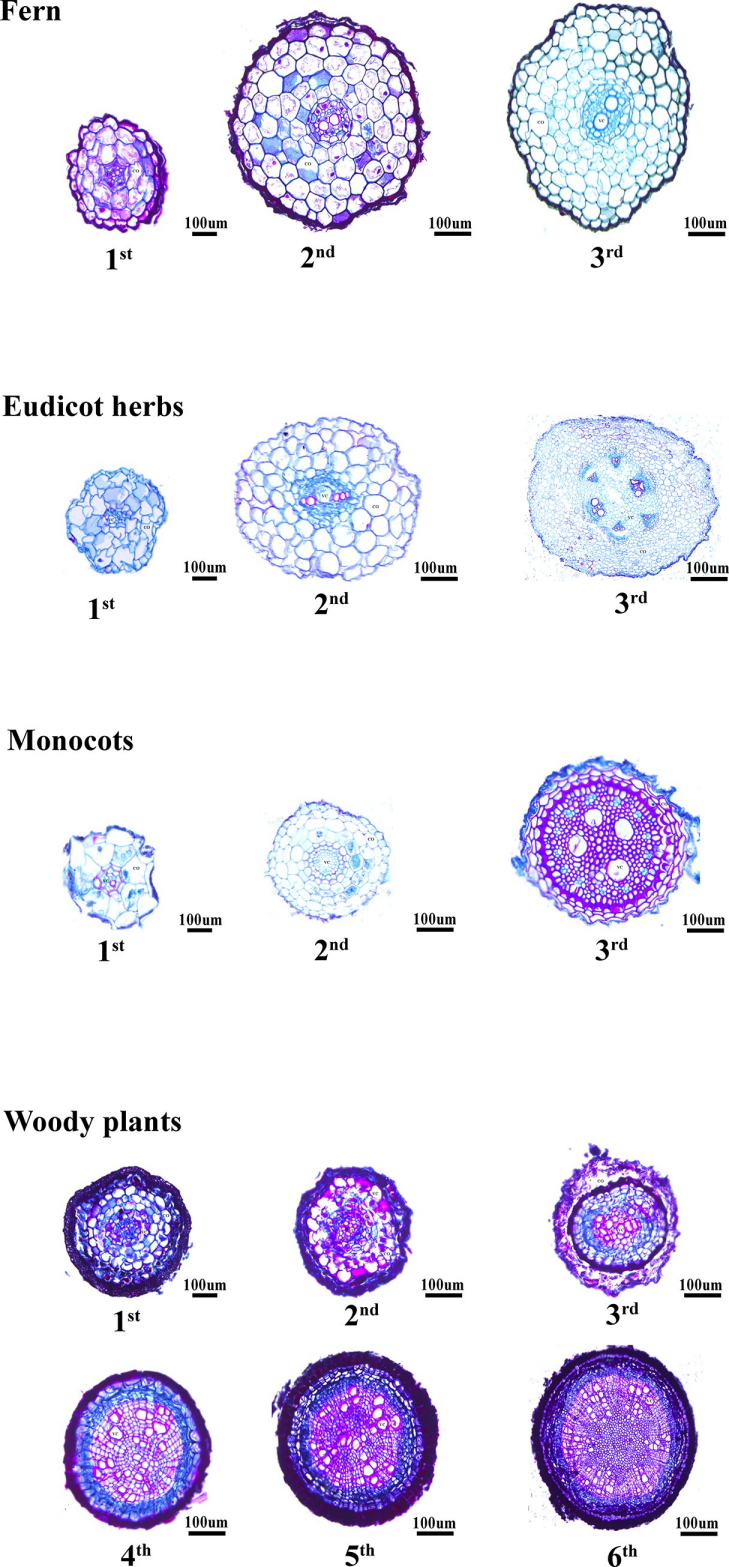
Root cross-sections of 4 species. Fern: *Onoclea sensibilis*; *Acte*: Eudicot herbs *Actinostemma tenerum*; Monocots: *Elymus dahuricus*; Woody plant: *Betula platyphylla*. S: Stele; C: Cortex; P: Periderm. Multiple: Fern: 1^st^ order (×150); 2^nd^ order (×100); 3^rd^ order (×100). Monocots: 1^st^ order (×150); 2^nd^ order (×100); 3^rd^ order (×100). Herbaceous eudicot: 1^st^ order (×150); 2^nd^ order (×100); 3^rd^ order (×100). Woody plants: 1^st^ order (×150); 2^nd^ order (×100); 3^rd^ order (×100); 4^th^ order (×100); 5^th^ order (×100); 6^th^ order (×100).

The lower-order roots of eudicot herbs, monocots and woody plants as well as ferns [23] apparently lack a Casparian strip or periderm (Fig 5). In the absence of a Casparian strip, these roots mainly control absorption and axial transport by controlling cortical thickness [6, 7], leading to a high correlation between the cortex and the stele diameter. In contrast, higher-order roots with a Casparian strip or periderm can control radial transport through these two structures [22, 25], which leads to a low correlation between the cortex and stele diameter. Additionally, this structural difference results in a much lower correlation between the cortex and root diameter but a higher correlation between the cortex and stele to diameter ratio in woody plants than in other groups.

The woody plants in this study have relatively small fine root diameters. There may be several factors contributing to this phenomenon. First, the plants in the study were all temperate plants, and temperate plants have significantly smaller root diameter than tropical plants [1]. Recent studies have found that root diameter, especially apical diameter, may differ substantially between tropical and temperate [32, 33] species. Second, the diameter of some gymnosperms (eg. *Pinus korainsis*) in this area is higher than the diameter of the fine roots of angiosperms [34] but the woody plants in this study are all angiosperms. These are all possible reasons for the smaller diameter of the fine roots.

### 4.2 Variation in traits

Remarkable variation was observed within each group. There were 3 factors contribute to the phenomenon. First, the number of root order were various within groups. For example, in 51 eudicot herbs species, 7.8% had 5 orders; 27.5% had 4 orders; 51.0% had 3 orders; 11.8% had 2 orders; and 1 species (*Actinostemma tenerum* Griff.) had only 1 order. It was a major resource of variation within group. Despite these differences, the highest order always had the largest stele diameter, so the greatest variation was in the middle orders. This may imply that the 1^st^ and highest order were similar among species; the only difference was how many orders exist between the 1^st^ and the highest order. There was an analogous situation in groups. tend to form finer diameters Second, some traits that were influenced by the environment also contribute to the variation. The root diameter of some trees is conserved [35]. But the environment of plants has a significant impact on their anatomical structure [36–40]. In monocots, the anatomical structures of hydrophytes and non-hydrophytes were divided into two categories: hydrophytic monocots exhibit fewer root orders with a large difference between different orders, whereas non-hydrophytic monocots show the opposite trend.

Furthermore, the transport process in the xylem is largely a physical process, and the transport function of the xylem cell can be accurately identified based on quantitative characteristics such as xylem cell number and diameter [41].

However, in contrast to those of the xylem, the function of the cortex is performed by living cells, and is influenced by many factors. These factors include the type and number of mycorrhizal fungi, the characteristics of the exodermal and endodermal Casparian strip and the number of passage cells (In most angiosperms, there are some unsuberized cells in endodermal of roots were called “passage cells” [42]); even the characteristics and number of plasmodesmata and channel proteins can significantly influence the function of the cortex [43–48]. These structures also differ markedly within plant groups, particularly in herbaceous eudicot and woody plants (Fig 5) (e.g., [49], leading to highly complex cortical thickness patterns within a given plant group.

### 4.3 Boundary division of fine-root function

Fine roots are generally defined according to diameter (with 1, 2 or 5 mm taken as the boundary between fine and coarse root) [2, 15, 50, 51], but this boundary between fine and coarse roots is thought to be arbitrary by researchers [2]. Furthermore, many people believe that fine roots can be functionally divided into two components: fine roots that are mainly responsible for absorption and fine roots that are mainly responsible for transport [15, 23, 52, 53]. From this perspective, it appears that plant roots can be divided into three categories: absorbing roots, transport roots, and coarse roots, of which the absorbing roots and transport roots constitute the traditional category of fine roots [2, 15]. In fine roots, the characteristics of the cortex and stele (including the xylem cell) may reflect the two functions of absorption and transport [7, 11]. It has been said to be reliable that the absorbing roots and transport roots can be divided according to the features of cortex and stele [15]. From lower to higher orders, the function of fine roots changes from absorption to transport [15].

**Fig 6:**
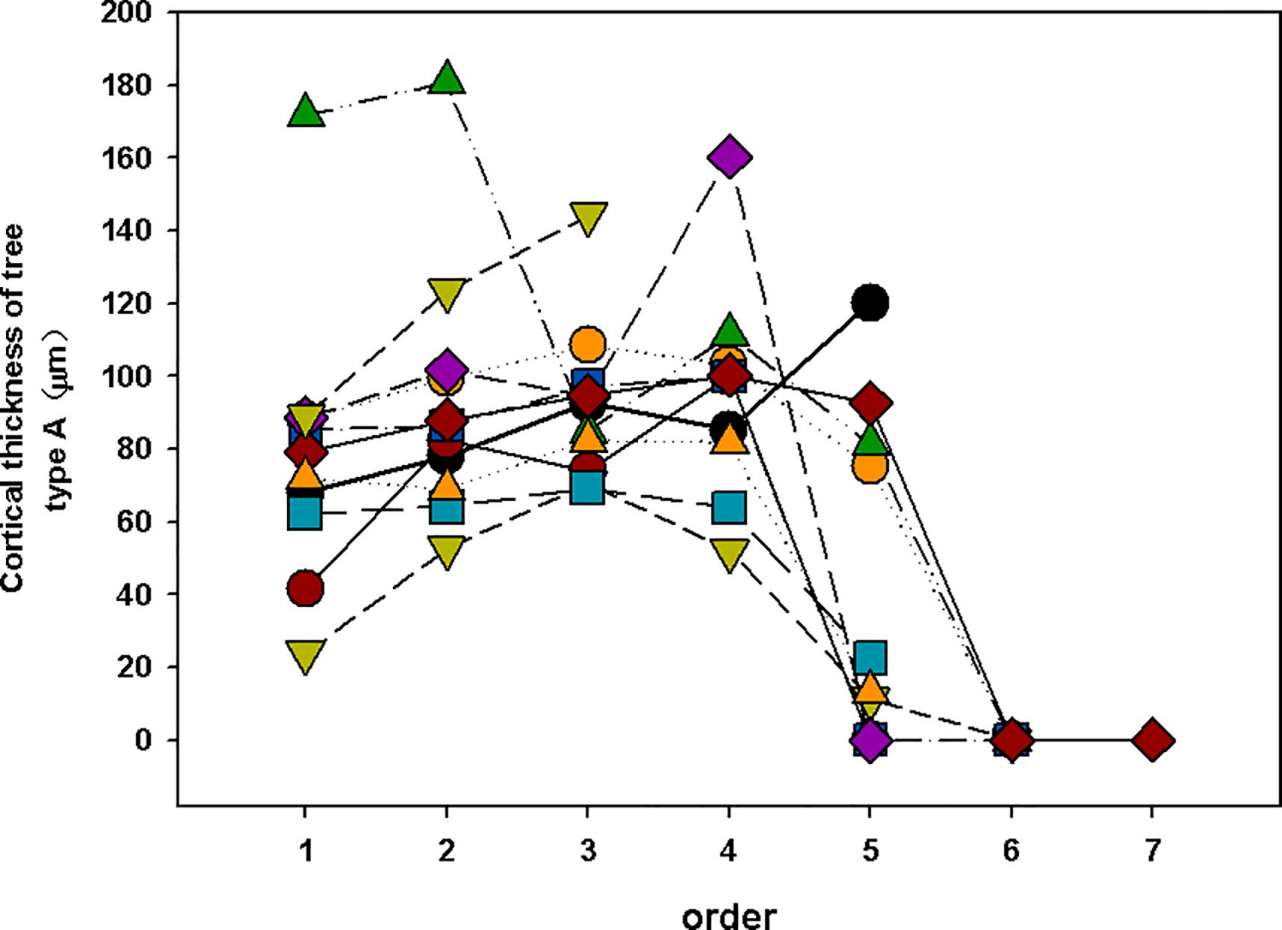
Cortical thickness of tree (type A). Type A: The cortex thickness increases in the first 3–4 order and then decline.

**Fig 7:**
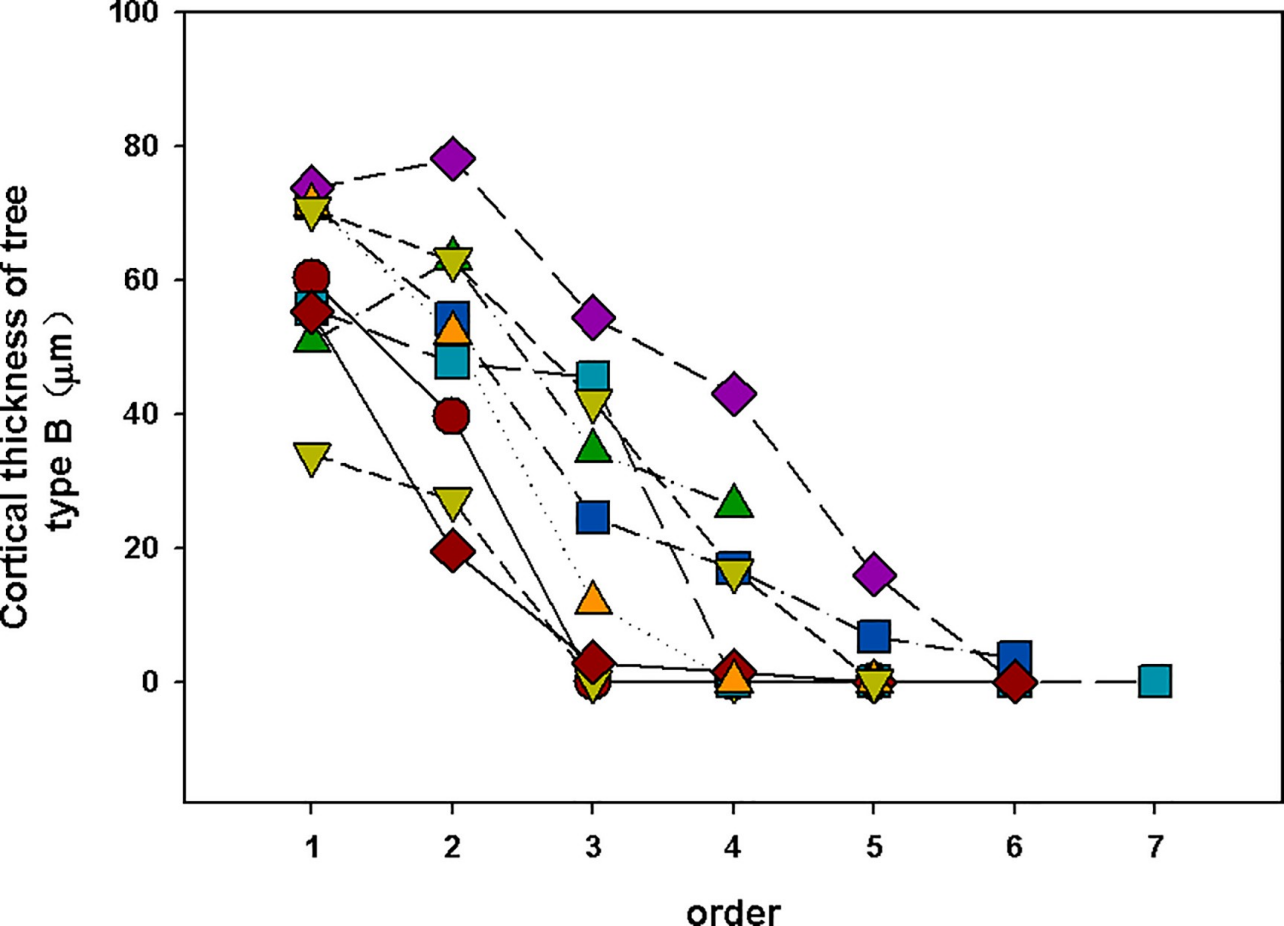
Cortical thickness of tree (type B). Type B: The thickest cortex is 1–2 order and then decline continuously.

**Fig 8:**
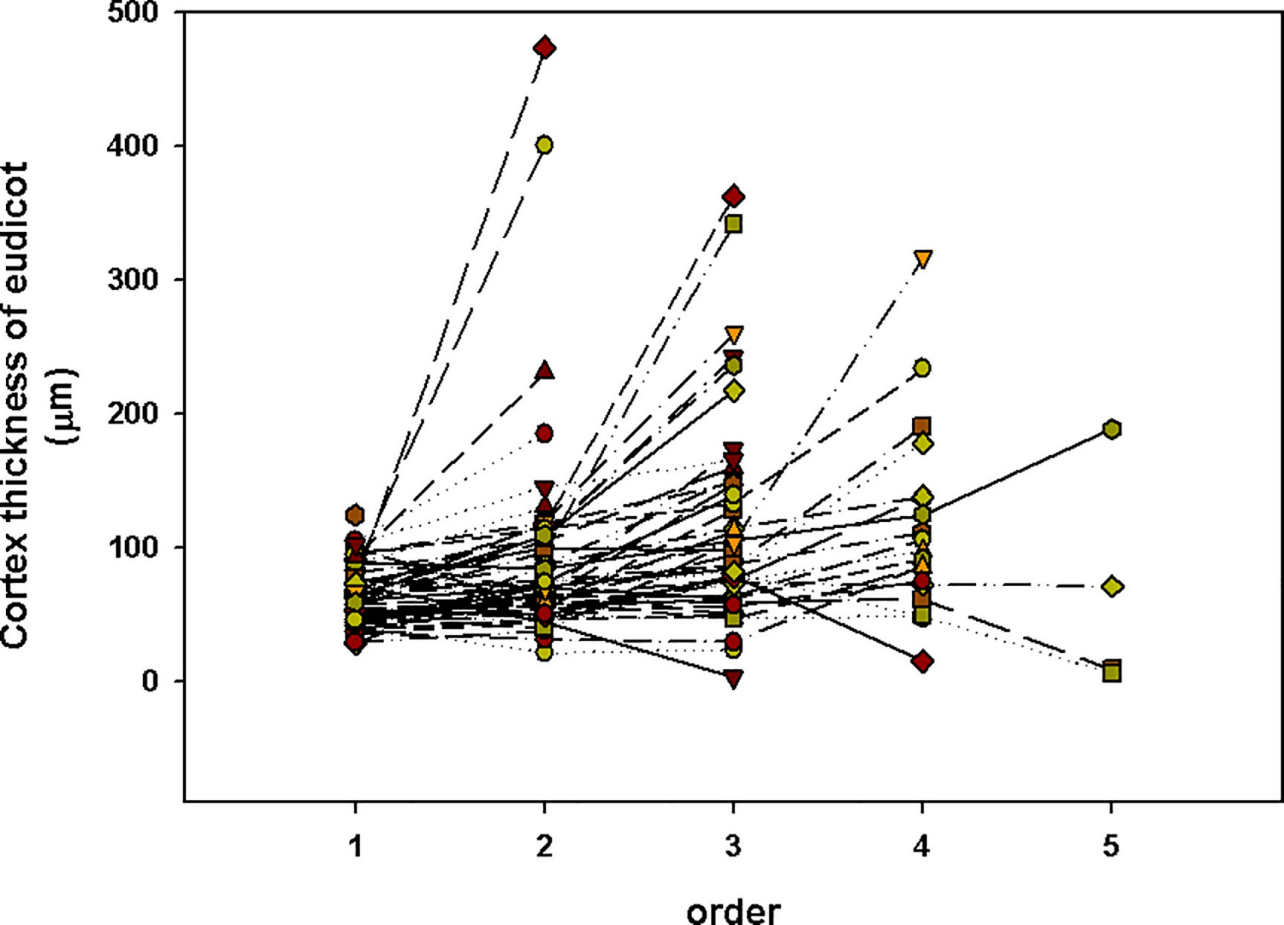
Cortex thickness of eudicot.

**Fig 9:**
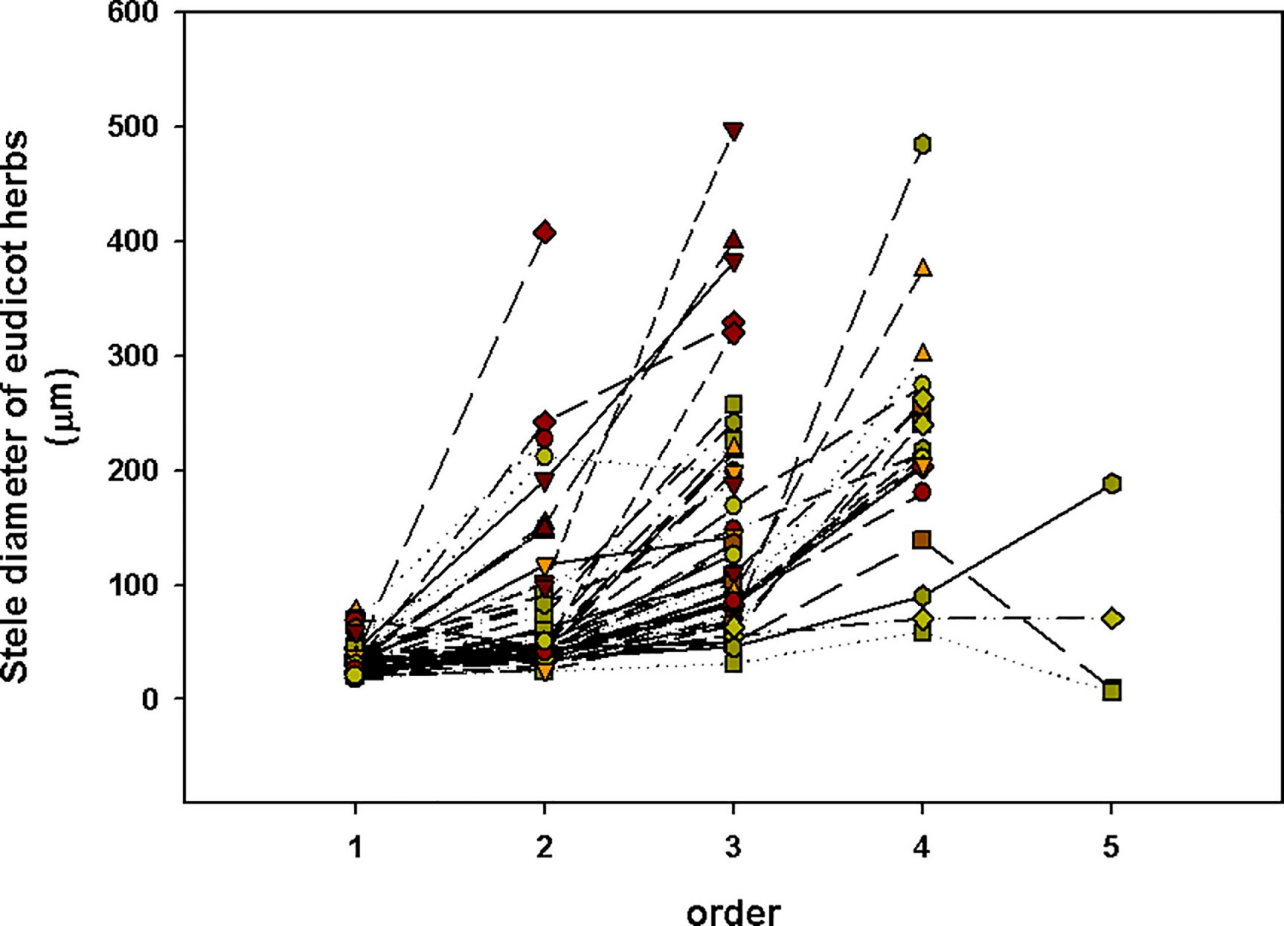
Stele diameter of eudicot herbs.

Here, there was boundary between lower-order (absorbing roots) and higher-order roots (transport roots). Such as there were no significant difference among 1^st^ to 4^th^ order in cortical thickness in woody plants, which implies that the 1^st^ to 4^th^ order of woody plants can be called “lower-order”. We think there are some reasons that contribute to the phenomenon of “cortex does not decline until the 5th order roots”. First, some species show a very different pattern. Such as *Menispermum dauricum*, a vine, has thicker cortex in the higher order. Second, the cortex of some species will increase in the 3–4 order (tape A) while the thickest cortex of other species is 1–2 order (type B) (Figs 6 and 7). That means the average cortex does not decline until the 5^th^ order roots in all woody plants. The cortex of eudicot herbs varies widely among species (Figs 8 and 9). However, a common feature of eudicot herbs is that the cortical thickness and stele diameter of the last order is significantly higher than that of the other orders. Thicker cortex in higher order roots may reduce their capability of radial transpor [11] and axial transport capacity greatly increases associated with decline in radial transport in the roots of herbaceous plants [29, 30]. Therefore, no matter how many orders, it is reasonable to take the last order as the transport root.

The boundary was different when using evidence from different traits. Most traits indicate that the first 2 or 3 root order were lower-order roots (absorbing roots) in eudicot herbs, and the 3^rd^- to 5^th^-order traits were higher-order roots (transport roots).

Most monocots had only two root orders; some had three root orders. In monocots, the boundary will shift with the number of root orders. The so-called lower-order roots (absorbing roots) will be of the 1^st^ order (only two root orders) or the first two orders (three root orders).

The boundary was different in ferns when using the evidence from different traits. Some traits, e.g., xylem cell diameter, indicate that the 1^st^ order was lower order (absorbing roots), but some other traits, e.g., stele diameter, cortical thickness and number of cortical cell layers, support the view that the 1^st^ to 2^nd^ orders were lower orders (absorbing roots) in ferns.

The situation was complex when we discuss the boundary in woody plants as there were conflicting observations. There were significant differences among 1^st^ to 5^th^ root order in the traits that indicate the root capacity to transport resources along the root. But there were no significant differences among 1^st^ to 4^th^ root order in the traits that indicate absorptive capacity. Presence of a periderm is a key trait associated with loss of absorption and loss of mycorrhizal colonization. The percentage of presence of a periderm increase with the orders. In most species (19 species, 83%) this percentage increase sharply to 20% to 67% in the 5^th^ order [7]. We believe the boundary should be the 4^th^ to 5^th^ order root. The first four or five orders were lower-order roots (absorbing roots) and the other roots were higher-order roots (transport roots) in woody plants.

Of course, these boundaries were not exact due to differences among species. Such as there was no “absorbing roots” and “transport roots” in Actinostemma tenerum Griff. because having only one root order. Furthermore, there were many differences within species. Such as: the coefficient of variation of stele diameter in the 5th root order of Spiraea chamaedryfolia up to 49%; in the 3rd root order of Betula platyphylla, 39% of roots in the stage of deutoxylem, 51% of roots in the stage of having vascular cambium, 10% of roots in the stage of having cork meristem.

## 5. Conclusions

The traits that indicate transport ability were always similar among groups, but the traits that indicate absorptive capacity differed greatly. The shift in function was the main factor that influences the anatomy of fine roots. Some traits show large variation within groups, but the variations in other traits were small. The traits indicate lower-order roots (absorbing roots) were the first 1 or 2 root order in ferns, the first 2 or 3 orders in eudicot herbs, the 1^st^ (only two root orders) or first two orders (more than three root orders) in monocots and the first four or five root orders in woody plants and the other roots were higher-order roots (transport roots).

## Supporting information

S1 Table Materials. (XLS)

S2 Table Data. (XLS)

S3 Table Raw data. (XLS)

S4 Table Test of normal distribution. (XLS)
